# Application of ^1^H NMR and HPLC-DAD in Metabolic Profiling of Extracts of *Lavandula angustifolia* and *Lavandula* × *intermedia* Cultivars

**DOI:** 10.3390/plants15020217

**Published:** 2026-01-10

**Authors:** Natalia Dobros, Katarzyna Zawada, Łukasz Woźniak, Katarzyna Paradowska

**Affiliations:** 1 Department of Organic and Physical Chemistry, Faculty of Pharmacy, Medical University of Warsaw, Banacha Str. 1, 02-097 Warsaw, Poland; katarzyna.zawada@wum.edu.pl (K.Z.); katarzyna.paradowska@wum.edu.pl (K.P.); 2 Department of Food Safety and Chemical Analysis, Institute of Agricultural and Food Biotechnology, State Research Institute, Rakowiecka Str. 36, 02-532 Warsaw, Poland; lukasz.wozniak@ibprs.pl

**Keywords:** *L*. *angustifolia*, *L*. × *intermedia*, polyphenolic compounds, ^1^H NMR profiling, HPLC-DAD, antioxidant activity

## Abstract

NMR spectroscopy enables the study of complex mixtures, including plant extracts. The interpretation of specific ranges of ^1^H NMR spectra allows for the determination of polyphenolic compound, sugar, amino acid, and fatty acid profiles. The main goal of ^1^H NMR analyses of plant extracts is to identify the unique “fingerprint” of the material being studied. The aim of this study was to determine the metabolomic profile and antioxidant activity of various *Lavandula angustifolia* (Betty’s Blue, Elizabeth, Hidcote, and Blue Mountain White) and *Lavandula* × *intermedia* cultivars (Alba, Grosso, and Gros Bleu) grown in Poland. Modern green chemistry extraction methods (supercritical fluid extraction (SFE) and ultrasound-assisted extraction (UAE)) were used to prepare the lipophilic and hydrophilic extracts, respectively. The secondary metabolite profiles were determined using the diagnostic signals from ^1^H NMR and HPLC-DAD analyses. These metabolomic profiles were used to illustrate the differences between the different lavender and lavandin cultivars. The HPLC-DAD analysis revealed that both lavender species have similar polyphenolic profiles but different levels of individual compounds. The extracts from *L. angustifolia* were characterized by higher phenolic acid and flavonoid contents, while the extracts from *L.* × *intermedia* had a higher coumarin content. Diagnostic ^1^H NMR signals can be used to verify the authenticity and origin of plant extracts, and identify directions for further research, providing a basis for applications such as in cosmetics.

## 1. Introduction

Lavender is a perennial plant cultivated in many countries in Europe, North Africa, Southwest Asia, and North America. Among the species cultivated in Poland, the dominant species are lavender (*Lavandula angustifolia*) and lavandin (*Lavandula* × *intermedia*), a hybrid of *Lavandula angustifolia* and *Lavandula latifolia*. The medicinal properties of lavender have been known since ancient times. In traditional medicine, lavender is used to treat nervous tension, difficulty sleeping, and gastrointestinal ailments [1]. Until now, the scientific research has focused primarily on the components of the essential oil [2,3] and its use as an antiseptic, which accelerates the healing of wounds, burns, and skin irritation.

However, some components of the essential oil can cause allergic reactions [4]. Therefore, there has been an increase in studies on alcohol–water extracts of lavender flowers, which could be used on people with known hypersensitivity to the oil’s components. Alcoholic–aqueous extracts of lavender flowers and leaves exhibit anti-inflammatory [5,6], antioxidant [7,8,9], antispasmodic [10], and antibacterial properties [11] due to their rich chemical composition.

An important technique for identifying plant secondary metabolites is high-performance liquid chromatography (HPLC) and ^1^H NMR spectroscopy, which are widely used in the pharmacological field. The high resolution of NMR spectroscopy allows for the study of complex mixtures such as plant extracts, which contain dozens of chemical substances. ^1^H NMR spectra enable the determination of sugar, amino acid, fatty acid, and polyphenolic compound profiles. Furthermore, NMR allows for metabolite profile analysis without the need for complex, multi-step separation of the mixture into individual fractions and compounds. In addition, the sample is not destroyed during analysis and can be used for further analysis. The main goal for using spectral methods such as ^1^H NMR in the analysis of plant extracts is to identify the unique “fingerprint” of the tested material [12,13]. The European Commission recognizes “NMR fingerprinting” as a method for verifying the authenticity and origin of plant materials or food [14]. This type of research can aid the EFSA in documenting health claims for plant products, enabling their promotion as health-promoting products. NMR analysis is used in the development of crucial procedures for the standardization of selected plant materials and extracts, which is crucial for in vivo testing. Often, divergent in vivo test results are the result of varying profiles of biologically active compounds.

The profile of polyphenolic compounds is characterized by significant variability depending on species, cultivar, geographic origin, climatic conditions, and extraction techniques [15,16]. Therefore, the aim of this study was to perform metabolomic profiling and antioxidant activity of various cultivars of *Lavandula angustifolia* (Betty’s Blue, Elizabeth, Hidcote, and Blue Mountain White) and *Lavandula* × *intermedia* (Alba, Grosso, and Gros Bleu) grown in Poland. Extracts were prepared using modern green chemistry extraction techniques. Ultrasound-assisted extraction (UAE) was used for the extraction of polar compounds, while supercritical fluid extraction (SFE) was used for the extraction of lipophilic compounds. The metabolomic profiles of the extracts were determined based on an analysis of diagnostic signals from ^1^H NMR spectroscopy and HPLC. To our knowledge, this is the first study investigating 70% methanol (*v*/*v*) and CO_2_ extracts of the Betty’s Blue, Elizabeth, Blue Mountain White, and Gros Bleu cultivars. The results provide important information regarding the polyphenolic profiles of lavender and lavandin cultivars grown in Poland. The diagnostic ^1^H NMR signals that can distinguish between cultivars and extraction methods can provide a basis for further biological (in vivo) studies, quality control studies, and the development of authentication methods for extracts.

## 2. Results and Discussion

### 2.1. Determination of Phenolic Compounds

#### 2.1.1. Determination of Total Phenolic Compound Content Using UV-Vis Method

Among the analysed extraction methods, the extracts obtained using ultrasound-assisted extraction had higher total polyphenol contents than the CO_2_ extracts (Table 1). The analysis of variance for all the cultivars showed statistically significant differences at the level of *p* < 0.05 between the 70% MeOH and CO_2_ extracts. For both types of extracts of *L*. *angustifolia* cultivars, the Betty’s Blue and Elizabeth cultivars had the highest total polyphenolic contents, followed closely by the Blue Mountain White and Hidcote cultivars. Among the 70% MeOH extracts of the *L*. × *intermedia* cultivars, the Alba cultivar had the highest total polyphenol content, followed closely by the Gros Bleu and Grosso cultivars. Among the CO_2_ extracts, the Gros Bleu cultivar had a lower total polyphenolic content than the Grosso cultivar.

The total polyphenol contents in the 70% MeOH extracts are much higher than those obtained by Bajalan et al. [15]. The authors examined 30 different populations of *L*. × *intermedia* occurring in western Iran. For methanol extracts, they obtained total polyphenol contents ranging from 0.31 to 1.05 mg_GAE/g_d.m. The significant differences in the content of polyphenolic compounds may be due to the different extraction and analysis methods, or diverse geographical origins, and thus different environmental and climatic conditions. Considerably lower total polyphenol contents were also reported by Adaszyńska-Skwirzyńska and Dzięcioł [17]. They found that the 70% MeOH extract of flowers of the *L*. *angustifolia* Ellagance Purple cultivar contained 1.12 mg_GAE/g_d.m., and the Blue River cultivar extract contained 1.13 mg_GAE/g_d.m. In the study by Hawrył et al. [7], the total polyphenol content in the 100% MeOH extract of the *L*. *angustifolia* Atropurpurea cultivar was 1.46 mg_GAE/mL (which corresponds to 72.9 mg_GAE/g_d.m.) and 1.98 mg_GAE/mL (98.9 mg_GAE/g_d.m.) in the *L*. *angustifolia* Rosea extract. This large discrepancy in the results may be caused by differences in the extraction procedure (different methods, extraction times, and temperatures) and in the chemical composition of the cultivars used.

Similarly, for the supercritical extracts, the obtained total polyphenol content is significantly higher than reported by Tyśkiewicz et al. [18] for Bulgarian lavender (*Lavandula angustifolia*) flowers, which was in the range of 0.28–0.94 mg_GAE/g of dried plant material. However, it should be noted that Tyśkiewicz et al. used only CO_2_ for supercritical extraction, with no co-solvent.

It should be noted that the Folin–Ciocalteu assay gives the approximation of phenolic content, as it is not specific to this class of compounds and could be influenced by the presence of antioxidants from other classes [19]. However, as in plants, phenolic compounds are usually the most abundant antioxidants, so in most such cases, this assay gives a rough approximation of total phenolic content, and it was taken as such also in this work.

#### 2.1.2. Determination of Phenolic Compounds Using HPLC Method

**Figure 1:**
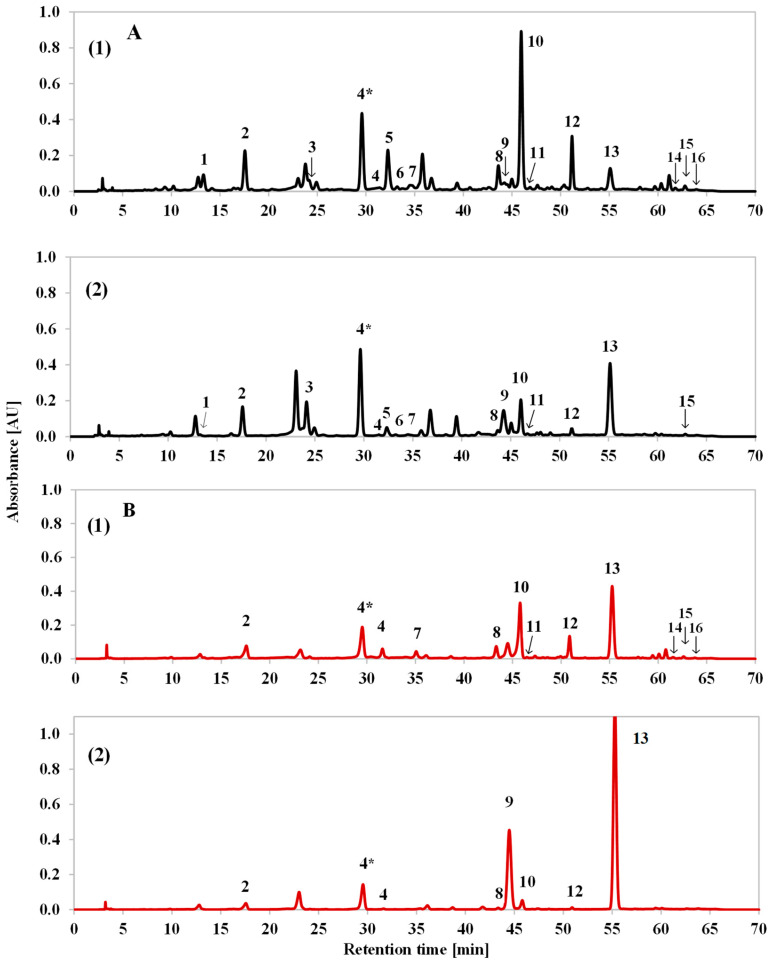
HPLC chromatograms at 330 nm for 70% MeOH extracts (**A**) and CO_2_ extracts (**B**) of *L*. *angustifolia* Betty’s Blue cultivar (**1**) and *L*. × *intermedia* Alba cultivar (**2**). 1—chlorogenic acid; 2—caffeic acid; 3—vanillin; 4*—ferulic acid glucoside; 4—ferulic acid; 5—ellagic acid; 6—rutin; 7—isoquercitrin; 8—hesperidin; 9—coumarin; 10—rosmarinic acid; 11—fisetin; 12—morin; 13—herniarin; 14—formononetin; 15—naringenin; 16—apigenin.

A qualitative analysis of the *L. angustifolia* and *L.* × *intermedia* extracts was performed by comparing the retention times of the peaks and their UV-Vis spectra with those obtained for the reference substances. The chromatogram at 330 nm for the mixture of standards is presented in Figure S1. The HPLC chromatograms for both types of extracts of the cultivars with the highest total polyphenol contents, Betty’s Blue (*L*. *angustifolia*) and Alba (*L*. × *intermedia*), are presented in Figure 1.

For both lavender species (*L*. *angustifolia* Betty’s Blue cultivar and *L*. × *intermedia* Alba cultivar), similar sets of polyphenolic compounds were obtained (Figure 1). The observed differences in peak intensities resulted from the different contents of compounds in the different cultivars and extracts. Phenolic acids, flavonoids, and coumarins were identified in both *L*. *angustifolia* and *L*. × *intermedia* extracts. The hydroxycinnamic acids chlorogenic acid (No. 1), caffeic acid (No. 2), ferulic acid (No. 4), and rosmarinic acid (No. 10) and the hydroxybenzoic acid ellagic acid (No. 5) were found. The most numerous groups of flavonoids were flavonols (rutin (No. 6), isoquercitrin (No. 7), fisetin (No. 11), and morin (No. 12)) and flavanones (hesperidin (No. 8) and naringenin (No. 15)). In addition, an isoflavone (formononetin (No. 14)), flavone (apigenin (No. 16)), phenolic aldehyde (vanillin (No. 3)), and two coumarins (coumarin (No. 9) and herniarin (No. 13)) were detected. Additionally, UHPLC-HRMS analysis was used to assign the compound marked with the symbol 4* due to a lack of a reference substance. The compound corresponding to the peak recorded on the chromatograms between 29 and 30 min was determined to be ferulic acid glucoside. The identification was based on the presence of [2M-H]^−^ ions at *m*/*z* 711.2132 and [M-H]^−^ at *m*/*z* 355.1034, as well as fragment ions at *m*/*z* 193.0513 and 149.0612, corresponding to the ferulic acid moiety after the loss of the glucoside part (162 Da) and the carboxyl group (44 Da). All of the identified masses matched the theoretical values within 1 ppm.

Rosmarinic acid, which is considered a chemotaxonomic marker in the *Lamiaceae* family, was confirmed to be the most common compound in lavender extracts by numerous scientific studies [11,17,20,21,22]. Caffeic acid and its derivatives were also frequently detected, but they were present in much smaller amounts [7,9,11,17,20,21,23,24]. The presence of ferulic acid glucoside in different types of extracts was also confirmed. It has been found in a 50% EtOH extract of *L*. *angustifolia* flowers and leaves [25] and an 80% MeOH extract of *L*. *angustifolia* Lady leaves [9]. It was also detected in plant material of *L. angustifolia* and the *L.* × *intermedia* Grosso cultivar remaining after distillation of the essential oil, which was then macerated using 50% EtOH [26]. Moreover, Areias et al. [27] identified coumarin and herniarin in ethanol extracts, while Najafian et al. [28] found coumarin in methanol extracts, and Jerković et al. [29] detected the presence of in supercritical carbon dioxide extracts of *Lavandula angustifolia* Mill. flowers. Numerous studies also confirmed the presence of other compounds, which were present in smaller amounts, such as chlorogenic acid [7,20,22,28], ferulic acid [9,11,20,23,30], vanillin [23,28], rutin [11,21,24], hesperidin, fisetin, formononetin [24], naringenin [21,24], and apigenin [7,17,20,21]. However, there are no reports documenting the presence of morin, which was one of the main compounds found in our *L*. *angustifolia* extracts, especially the Betty’s Blue extracts (Figure 1, Table 2). This highlights the variability in chemical profiles between the cultivars analysed so far. Therefore, based on our studies, the presence of morin could be a parameter differentiating the Betty’s Blue cultivar from other *L*. *angustofolia* and *L*. × *intermedia* cultivars.

A quantitative analysis of the *L*. *angustifolia* and *L*. × *intermedia* extracts was performed for compounds representing each of the identified groups of polyphenolics: phenolic acids (rosmarinic acid and caffeic acid), flavonoids (morin), and coumarins (herniarin and coumarin) (Table 2). The contents of the individual compounds in the 70% MeOH and CO_2_ extracts are presented in mg/g of the dry extract (mg/g_d.e.). In addition, the content of ferulic acid glucoside, a dominant compound in most of the lavender and lavandin extracts, was calculated using a standard curve for ferulic acid and expressed as ferulic acid equivalents due to the lack of a reference substance. The analysis of variance showed significantly higher rosmarinic acid, ferulic acid glucoside, and morin contents in the 70% MeOH extracts compared to the CO_2_ extracts. On the other hand, the CO_2_ extracts had significantly higher coumarin and herniarin contents. Next, a detailed analysis of the polyphenolic compounds in each type of extract was performed.

The 70% MeOH extracts of the lavender species had similar caffeic acid contents (Table 2). The highest values for ferulic acid glucoside were obtained for the Blue Mountain White cultivar, followed by the Elizabeth, Hidcote, and Grosso cultivars. Significantly lower values were obtained for the remaining cultivars (Alba, Betty’s Blue, and Gros Bleu). The Betty’s Blue and Elizabeth cultivars had the highest rosmarinic acid and morin contents, while the Blue Mountain White, Alba, Grosso and Gros Bleu cultivars had significantly lower contents. On the other hand, the *L*. × *intermedia* extracts (Alba, Grosso, and Gros Bleu) had two-fold higher coumarin and herniarin contents compared to the *L*. *angustifolia* extracts.

Adaszyńska-Skwirzyńska and Dzięcioł [17] obtained a value of 5.57 mg of rosmarinic acid/g_d.e. for an 80% MeOH extract of the Ellagance Purple cultivar, which is similar to the value we obtained for the 70% MeOH extract of the Blue Mountain White cultivar (5.03 mg/g_d.e.). On the other hand, their rosmarinic acid content for the Blue River cultivar (2.13 mg/g_d.e.) was slightly lower than the content we obtained for the Gros Bleu cultivar (3.94 mg/g_d.e.). Regarding caffeic acid content, they found that the Ellagance Purple and Bleu River cultivars had significantly lower contents (0.12 and 0.08 mg/g_d.e., respectively) than the Blue Mountain White cultivar (2.05 mg/g_d.e.), which we found to have the lowest value. Lower values for caffeic acid (0.12 mg/g_d.e.) and rosmarinic acid (0.50 mg/g_d.e.) contents were also obtained by Zgórka and Głowniak [31] for a methanol extract of *L*. *angustifolia*.

The CO_2_ extracts (Table 2) contained a significant amount of coumarins (coumarin and herniarin) and small amounts of phenolic acids and morin. The Alba and Grosso cultivars had the highest coumarin and herniarin contents, with significantly lower values for the Blue Mountain White cultivar, followed by the Elizabeth, Hidcote, and Betty’s Blue cultivars. Significantly lower values were obtained for the Gros Bleu cultivar compared to the other cultivars, though they were still higher than those obtained by Jerković et al. [29] for supercritical extracts obtained without any co-solvent, which were up to 2.2 mg/g_d.e. for coumarin and up to 1.85 mg/g_d.e. for herniarin. Moreover, for the Gros Bleu cultivar, apart from coumarins, no other groups of compounds were identified. Among the phenolic acids in the CO_2_ extracts, ferulic acid glucoside dominated, with the highest content in the Elizabeth and Blue Mountain White cultivars. The Betty’s Blue and Elizabeth cultivars had the highest rosmarinic acid, morin, and caffeic acid contents. However, these values did not differ significantly from the values obtained for the other cultivars.

### 2.2. NMR Analysis

Individual chemical compounds were identified based on the chemical shift values for signals originating from characteristic protons. The HMDB (Human Metabolome Database) database and the work by Silverstein et al. [32] were used in the analysis.

In the first stage, ^1^H NMR spectra were recorded for the 70% MeOH and CO_2_ extracts of four *L*. *angustifolia* cultivars (Betty’s Blue, Elizabeth, Hidcote, and Blue Mountain White) and three *L*. × *intermedia* cultivars (Alba, Grosso, and Gros Bleu) (Figure S2). In the ^1^H NMR spectra of each cultivar, three ranges of proton signals were analysed: signals in the aliphatic region from 1.45 to 2.70 ppm (green frame), the sugar region from 3.45 to 4.60 ppm (red frame), and the aromatic region from 5.50 to 8.55 ppm (blue frame). The signal at the chemical shift value of δH = 3.30 ppm came from the solvent, which was deuterated methanol (CD_3_OD), while the signal at δH = 4.90 ppm came from water from the 70% MeOH used for extraction or, in the case of supercritical extraction, the co-solvent (75% EtOH). Regardless of the region and the cultivar, the ^1^H NMR spectra of the CO_2_ extracts (Figure S2B) had fewer resonance signals than the spectra for the 70% MeOH extracts (Figure S2A). The greatest difference was observed in the aliphatic region, where the CO_2_ extracts had no signals from sugar protons (red frame in Figure S2) due to this type of extraction not favouring the extraction of these strongly hydrophilic compounds. In the aromatic region in the spectra of the CO_2_ extracts, there was no signal from the acidic proton at δH = 8.50 ppm. In the 70% MeOH extracts, this signal most likely came from the formic acid used to acidify the 70% MeOH. The intensity of the remaining signals was also significantly lower in the spectra of the CO_2_ extracts compared to those for the 70% MeOH extracts.

Nevertheless, it was possible to distinguish between the two lavender species regardless of the extraction method used. In the spectra for the 70% MeOH extracts (Figure S2A) in the range from 1.45 to 2.70 ppm (green frame), the presence of signals at chemical shift values of δH = 1.95 and 2.50 ppm allowed us to distinguish *L*. *angustifolia* cultivars from *L*. × *intermedia* cultivars. In the spectra for the CO_2_ extracts (Figure S2B), the area that could differentiate *L*. × *intermedia* cultivars from *L*. *angustifolia* cultivars was the region of aromatic protons in the range of 6.20–8.00 ppm (blue frame) and the resonance signal occurring at δH = 3.90 ppm (red frame).

**Figure 2:**
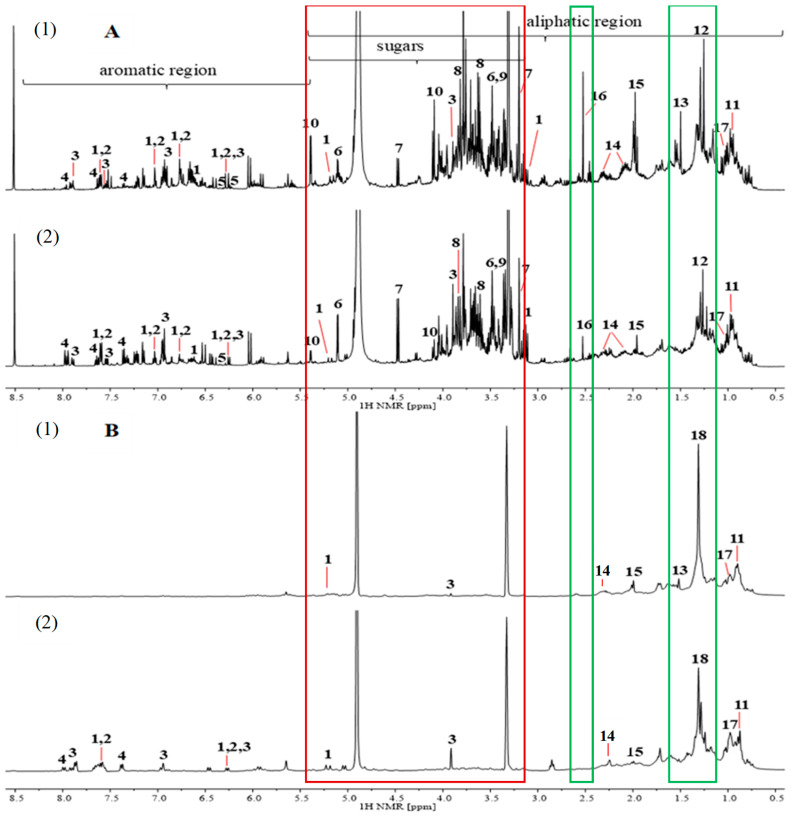
^1^H NMR spectra in the range from 0.50 to 8.50 ppm for 70% MeOH (**A**) and CO_2_ extracts (**B**) of *L*. *angustifolia* Betty’s Blue cultivar (**1**) and *L*. × *intermedia* Alba cultivar (**2**). 1—rosmarinic acid; 2—caffeic acid; 3—herniarin; 4—coumarin; 5—morin; 6—α-glucose; 7—β-glucose; 8—D-galactose; 9—L-rhamnose; 10—sucrose; 11—valine; 12—threonine; 13—alanine; 14—glutamine; 15—acetic acid; 16—pyruvic acid; 17—linolenic acid; 18—palmitic acid. Green frame—differences in the aliphatic region; red frame—differences in the sugar region.

In the subsequent stage of the study, a more detailed analysis of the composition of the cultivar with the highest polyphenol content in each lavender species was carried out. The ^1^H NMR spectra in the range from 0.50 to 8.50 ppm for the 70% MeOH and CO_2_ extracts of the Betty’s Blue (*L*. *angustifolia*) and Alba (*L*. × *intermedia*) cultivars are shown in Figure 2. Based on the results obtained by Koycheva et al. [33] and Wahyuni et al. [34], characteristic signals in the range from 0.90 ppm to 1.50 ppm that originated from protons in amino acids were assigned. A signal that could distinguish between the cultivars was a doublet originating from protons of the methyl group (-CH_3_) of alanine (No. 13, green frame). The signal at δH = 1.50 ppm was only visible in the spectra of the extracts of the Betty’s Blue cultivar. Protons of the methyl group of valine (No. 11) at δH = 0.85 ppm were present in both types of extracts for both cultivars, while the signal from threonine (No. 12, green frame) at δH = 1.30 ppm was only present in the 70% MeOH extracts. According to the HMDB database and the reports by Lupoae et al. [35] and Siudem et al. [36], the signal at δH = 1.31 ppm (No. 18, green frame) in the CO_2_ extract spectra for the Betty’s Blue and Alba cultivars came from the protons of the -(CH_2_)n- group of a fatty acid, most likely palmitic acid. On the other hand, the signal at δH = 0.97 ppm (No. 17) that was present in all the spectra (both types of extracts for both cultivars) came from the protons of the -CH=CH-CH_2_-CH_3_ group of linolenic acid [37].

In the aliphatic region from 1.95 ppm to 2.50 ppm, there was a singlet with a chemical shift of δH = 2.50 ppm (No. 16, green frame), which originated from protons in the CH_3_– group connected to the carbonyl carbon (C=O), could differentiate between the types of extracts: its intensity was higher in the spectra for the 70% MeOH extract of the Betty’s Blue cultivar (Figure 2A). According to the HMDB database, this signal could originate from pyruvic acid, which was confirmed by the ^1^H NMR analysis results of Koychev et al. [33] for a 50% MeOH extract of *L*. *angustifolia* obtained from a cell suspension culture. The signal at δH = 1.95 ppm (No. 15) was assigned to methyl protons of acetic acid. Similarly to pyruvic acid, this signal had a higher signal intensity in the spectra for the extracts of the Betty’s Blue cultivar. In turn, in both types of extract and both lavender cultivars, signals in the form of multiplets were observed at δH = 2.14 and 2.35 ppm (No. 14, green frame), indicating the presence of glutamine–glutamic acid amide.

In the ^1^H NMR spectra for the 70% MeOH extracts (Figure 2A), a range of signals (from 3.18 ppm to 5.40 ppm, red frame) corresponding to protons from the –CH and –CH_2_ groups originating from sugars was clearly visible, which were not present in the proton spectra for the CO_2_ extracts. Based on their chemical shift values, the presence of α-glucose (characteristic signal at δH = 5.10 and 3.45 ppm; No. 6) and the second anomer of this sugar, β-glucose (δH = 4.50 and 3.18 ppm; No. 7), was identified [34,38]. The signals located at the chemical shift values of δH = 5.40 and 4.15 ppm (No. 10) indicated the presence of sucrose; the shifts at δH = 3.85 and 3.65 ppm (No. 8) indicated the presence of D-galactose; and the signal at δH = 3.54 ppm (No. 9) indicated the presence of L-rhamnose [33,34]. In the range for sugar protons, a single signal (singlet) with a chemical shift value of δH = 3.90 ppm (No. 3) was detected, which came from the protons of the CH_3_-O-herniarin group; in both types of extracts, this signal’s intensity was higher in the spectra for the Alba cultivar compared to that of the Betty’s Blue cultivar.

**Figure 3:**
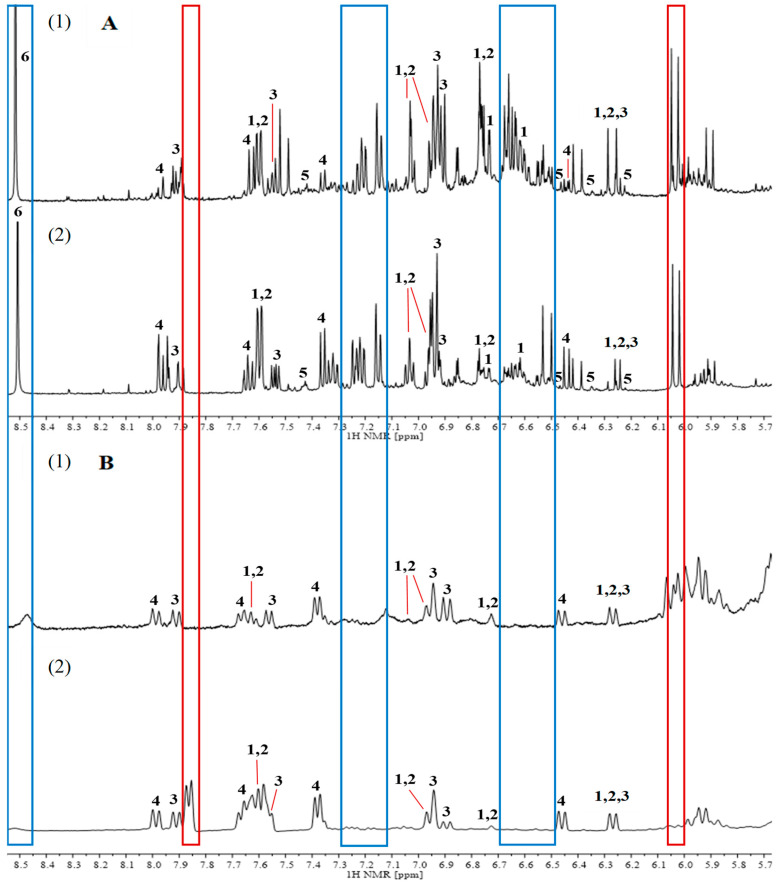
^1^H NMR spectra in the aromatic region from 5.80 to 8.55 ppm for 70% MeOH extracts (**A**) and CO_2_ extracts (**B**) of *L*. *angustifolia* Betty’s Blue cultivar (**1**) and *L*. × *intermedia* Alba cultivar (**2**). 1—rosmarinic acid; 2—caffeic acid; 3—herniarin; 4—coumarin; 5—morin; 6—acidic proton. Blue frame—differences between extraction methods; red frame—differences between cultivars for CO_2_ extracts.

In the aromatic region (Figure 3), the spectra of the 70% MeOH extracts were characterized by a greater number of resonance signals. Signals from protons of rosmarinic acid at δH = 6.63 ppm (No. 1) and morin at δH = 6.22, 6.35, and 6.46 ppm (No. 5), as well as signals at δH = 6.48–6.70, and 7.12–7.30 ppm (blue frame) were recorded for both cultivars, but only for the 70% MeOH extracts. In the spectrum of the CO_2_ extract, the doublet at the chemical shift value of δH = 6.05 ppm (red frame) was present only for the Betty’s Blue cultivar, whereas the doublet at δH = 7.85 ppm (red frame) only for the Alba cultivar. The doublets at δH = 7.57 and 6.25 ppm (No. 1 and 2) that were present in all the spectra (both extracts and both lavender cultivars) came from protons attached to carbon atoms, forming an unsaturated bond in the aliphatic chain of rosmarinic acid and caffeic acid molecules (-HC=CH-). The signals originating from protons attached to carbon atoms in the aromatic ring of herniarin occurred at chemical shift values of δH = 7.90, 7.52, 6.94, 6.91, and 6.25 ppm (No. 3). Moreover, based on the work of Ferreira et al. [39], the signals with chemical shifts equal to δH = 7.97, 7.65, 7.37, and 6.45 ppm (No. 4) were assigned to protons present in coumarin, which was confirmed to be present in the extracts by the HPLC analysis.

**Figure 4:**
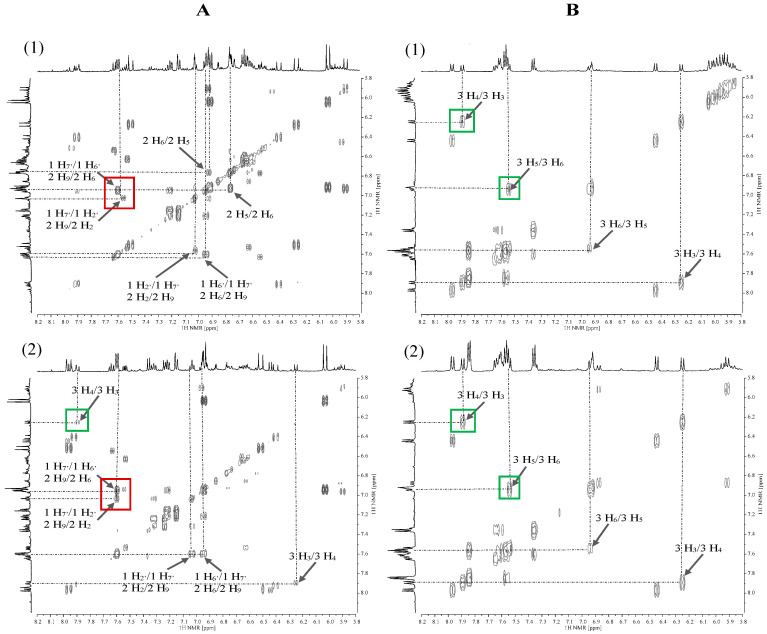
COSY correlation spectra in the aromatic region from 5.80 to 8.20 ppm for 70% MeOH extracts (**A**) and CO_2_ extracts (**B**) of *L*. *angustifolia* Betty’s Blue cultivar (**1**) and *L*. × *intermedia* Alba cultivar (**2**). 1—rosmarinic acid and 2—caffeic acid (red frame), 3—herniarin (green frame).

The next step after analysing the ^1^H NMR spectra was the interpretation of two-dimensional correlation spectra (2D NMR). COSY spectra were used to observe homonuclear correlations (^1^H–^1^H) between the nuclei of non-equivalent protons. In the spectra for 70% MeOH extracts of Betty’s Blue (Figure 4(A1)) and Alba (Figure 4(A2)) cultivars, correlation signals originating from conjugated protons of rosmarinic acid (No. 1) and caffeic acid (No. 2) occur (red frame), which correspond to a doublet at a chemical shift value of approximately δH = 7.56 ppm (1 H_7′_ and 2 H_9_, respectively) and a doublet of doublets at δH = 6.96 ppm (1 H_6′_ and 2 H_6_) and a doublet at δH = 7.06 ppm (1 H_2′_ and 2 H_2_). In the spectrum for 70% MeOH extract of the Alba cultivar, there is a correlation between herniarin protons (green frame) occurring as doublets at δH = 7.89 ppm (3 H_4_) and δH = 6.25 ppm (3 H_3_). In turn, in the COSY spectra for CO_2_ extracts of both the Betty’s Blue cultivar (Figure 4(B1)) and the Alba cultivar (Figure 4(B2)), correlation signals only from herniarin protons are present. The proton (3 H_4_) at δH = 7.89 ppm couples with the proton (3 H_3_) at δH = 6.25 ppm, and the proton (3 H_5_) at δH = 7.52 ppm couples with the proton (3 H_6_) at δH = 6.94 ppm.

**Figure 5:**
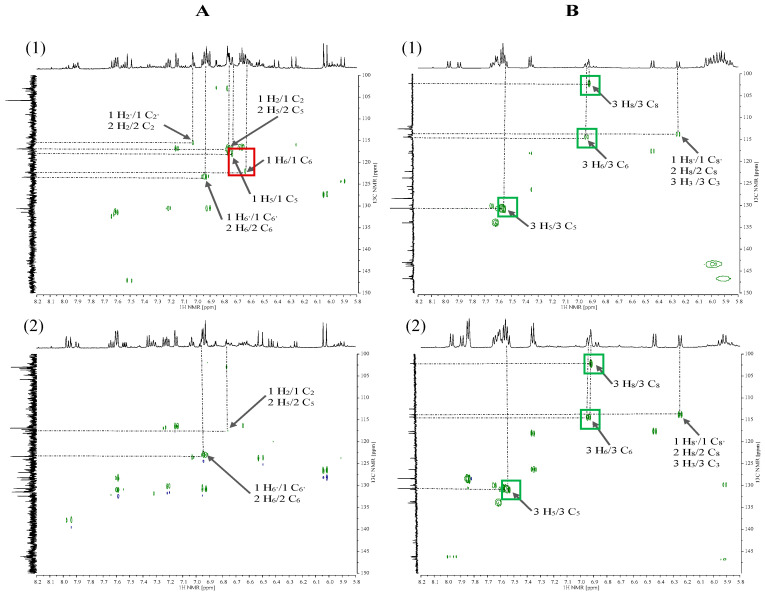
HSQC correlation spectra in the aromatic region for 70% MeOH extracts (**A**) and CO_2_ extracts (**B**) of *L*. *angustifolia* Betty’s Blue cultivar (**1**) and *L*. × *intermedia* Alba cultivar (**2**). 1—rosmarinic acid and 2—caffeic acid (red frame), 3—herniarin (green frame).

In the HSQC spectra, heteronuclear couplings are observed via a single bond between the ^1^H and ^13^C nuclei. In the spectrum for 70% MeOH extract of Betty’s Blue cultivar (Figure 5(A1)), the proton (1 H_6_) of the rosmarinic acid molecule, which corresponds to the signal visible at δH = 6.63 ppm, couples with the carbon atom (1 C_6_) giving a signal at approximately δC = 122.50 ppm, while the proton (1 H_5_) at δH = 6.73 ppm couples with the carbon atom (1 C_5_) at δC = 118.00 ppm (red frame). Correlation signals for the protons and carbons of rosmarinic acid and caffeic acid were also observed in the spectrum.

In the case of CO_2_ extracts (Figure 5(B1,B2)) for both lavender cultivars, the HSQC spectra show correlation signals from protons and carbon atoms of herniarin (green frame) observed at δH = 6.91 ppm (3 H_8_) and δC = 102.00 ppm (3 C_8_), δH = 6.94 ppm (3 H_6_) and δC = 114.50 ppm (3 C_6_) and δH = 7.52 ppm (3 H_5_) and δC = 131.00 ppm (3 C_5_). Additionally, for CO_2_ extracts, protons from herniarin (3 H_3_), rosmarinic acid (1 H_8′_) and caffeic acid (2 H_8_), whose signal occurs at δH = 6.25 ppm, couple with carbon atoms (3 C_3_, 1 C_8′_, 2 C_8_), giving a signal with a chemical shift of δC = 114.00 ppm.

**Figure 6:**
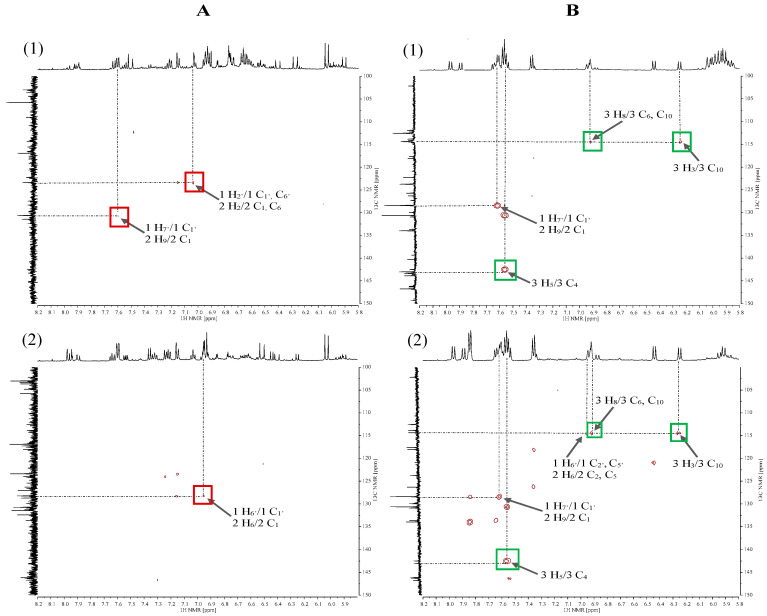
HMBC correlation spectra in the aromatic region from 5.80 to 8.20 ppm for 70% MeOH extracts (**A**) and CO_2_ extracts (**B**) of *L*. *angustifolia* Betty’s Blue cultivar (**1**) and *L*. × *intermedia* Alba cultivar (**2**). 1—rosmarinic acid and 2—caffeic acid (red frame), 3—herniarin (green frame).

HMBC spectra were used to observe heteronuclear coupling between ^1^H and ^13^C nuclei separated by 2 or 3 bonds. Couplings between protons and carbon atoms occurring in the rosmarinic acid and caffeic acid molecules (red frame) were observed in the spectra for 70% MeOH extracts recorded for Betty’s Blue (Figure 6(A1)) and Alba (Figure 6(A2)) cultivars. The correlation signals for the Betty’s Blue cultivar are present between the doublet with the chemical shift value of δH = 7.02 ppm (1 H_2′_ for rosmarinic acid and 2 H_2_ for caffeic acid) and the signals of carbon atoms (1 C_1′_, 1 C_6′_ and 2 C_1_, 2 C_6_) at the value of δC = 123.48 ppm and the doublet at δH = 7.61 ppm (1 H_7′_ and 2 H_9_) and the signal from carbon atoms (1 C_1′_ and 2 C_1_) at the value of δC = 130.89 ppm. In turn, in the spectrum for the Alba cultivar, the proton for rosmarinic acid (1 H_6′_) and caffeic acid (2 H_6_) at δH = 6.95 ppm couples with the carbon atom (1 C_1′_) and (2 C_1_) giving a signal recorded at the value of δc = 128.29 ppm.

The spectra recorded for CO_2_ extracts of both lavender cultivars (Figure 6(B1,B2)) are dominated by correlation signals originating from couplings between protons and carbon atoms of herniarin (green frame). The proton (3 H_3_) giving a signal occurring as a doublet at δH = 6.24 ppm couples with the carbon atom (3 C_10_) giving a signal at δC = 114.39 ppm, the proton (3 H_8_) at δH = 6.91 ppm with carbon atoms (3 C_6_ and 3 C_10_) at δC = 114.39 ppm, and the proton (3 H_5_) at δH = 7.55 ppm with the carbon atom (3 C_4_) at δC = 143.00 ppm. The spectra for CO_2_ extracts also contain correlation signals originating from the coupling of protons and carbon atoms of rosmarinic acid and caffeic acid.

In summary, the analysis of the NMR spectra for the two cultivars belonging to different lavender species allowed for the identification of differences in the chemical composition of the extracts in both the aliphatic and aromatic regions. The differences included different signals and signal intensities. One of these differences was a doublet of the protons of the methyl group of alanine that was only present in the aliphatic region in the ^1^H NMR spectra of the Betty’s Blue cultivar extracts. Signals distinguishing the two types of extracts were also identified. The signals from threonine (δH = 1.30 ppm), pyruvic acid (δH = 2.50 ppm), and sugars were only present in the 70% MeOH extracts, whereas the signal from palmitic acid (δH = 1.31 ppm) was only in the CO_2_ extracts. In the aromatic region, in the CO_2_ extracts, there were two signals that differentiated the two cultivars (δH = 6.05 ppm for the Betty’s Blue cultivar and δH = 7.85 ppm for the Alba cultivar). Nevertheless, the spectra recorded for the CO_2_ extracts had fewer resonance signals than the spectra for the 70% MeOH extracts due to the lack of signals characteristic of the protons of water-soluble compounds. In the aromatic region, the signals for polyphenolic compounds were characterized by a much lower intensity, and in the aliphatic region, no signals for sugars were recorded. On the other hand, the signals in the spectra recorded for the CO_2_ extracts in the aliphatic region were due to the presence of fatty acids. Two-dimensional NMR spectroscopy also highlighted differences between the two types of extracts. In most cases, correlation signals from protons and carbons present in the rosmarinic acid and caffeic acid molecules were observed in the correlation spectra recorded for the 70% MeOH extracts, while signals from the herniarin molecule were observed in the spectra recorded for the CO_2_ extracts. Therefore, NMR correlation analysis confirmed that rosmarinic acid may be the potential marker for the Betty’s Blue cultivar, and herniarin for the Alba cultivar.

### 2.3. Antioxidant Activity

The antioxidant activity of the extracts was determined using FRAP and DPPH methods, which differ in their reaction mechanisms. The FRAP assay is based on the reduction in oxidant metal ions by single electron donation. In contrast, the DPPH assay combines electron transfer and hydrogen atom transfer mechanisms, leading to radical reduction. The results of antioxidant activity analyses were expressed as Trolox equivalents (TE) and were presented in Table 3.

For the *L*. *angustifolia* extracts, the highest antioxidant properties, in both FRAP and DPPH assays, were obtained for the Betty’s Blue and Elizabeth cultivars, and slightly lower for Blue Mountain White and Hidcote. Among the *L*. × *intermedia* cultivars, the highest antioxidant properties were observed for the Alba cultivar, and slightly lower for the Gros Bleu and Grosso cultivars. Ultrasound-assisted extraction using a polar solvent contributed to more effective extraction of polyphenolic compounds with antioxidant activity. Multiple regression analysis showed that the antioxidant activity of 70% MeOH extracts was related to the content of rosmarinic acid and caffeic acid.

Ahn et al. [9] obtained 9 µmol_TE/g_d.m. in the DPPH assay for 80% MeOH extracts for the Hidcote cultivar, and 23 µmol_TE/g_d.m. in the FRAP assay, while for the Lady cultivar 14 and 30 µmol_TE/g_d.m., respectively. Our values for 70% MeOH extracts were significantly higher, which may be due to the use of ultrasound, whereas the authors used shaking. On the other hand Celik et al. [21] obtained a significantly higher value of 390 µmol_TE/g_d.m. than those presented in our study. Authors used the CUPRAC method, which like the FRAP method, is based on chemical reactions according to the SET mechanism.

### 2.4. Statistical Analysis

**Figure 7:**
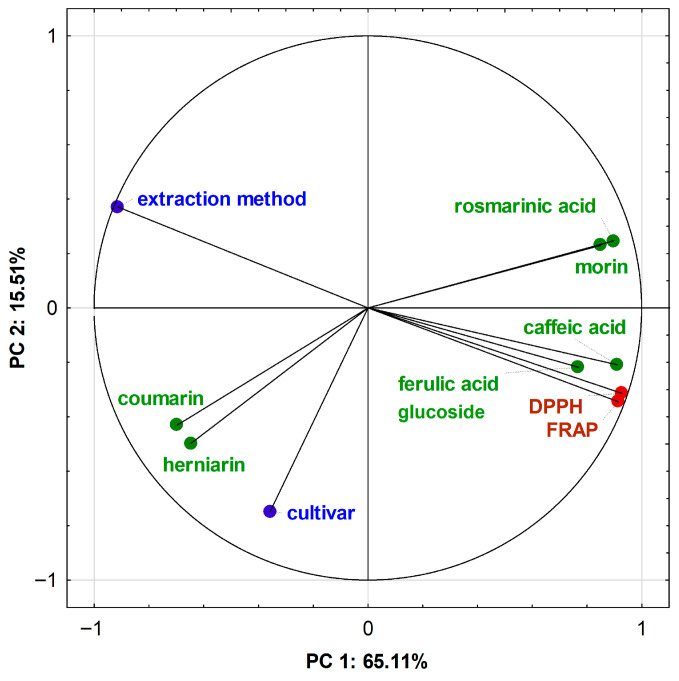
PCA correlation circle for the HPLC and antioxidant data for *L*. *angustifolia* and *L*. × *intermedia* extracts.

A correlation circle was used to visualise the correlation between the principal components (PC) and the original variables for the *L*. *angustifolia* and *L*. × *intermedia* extracts (Figure 7). Principal component analysis (PCA) analysis showed that the first two principal components (PC 1 and PC 2) explained 80.62% of the variability in the data. The first principal component (PC 1) accounted for 65.11% of the total variance, while the second principal component (PC 2) accounted for 15.51%.

The first principal component is most strongly correlated with the antioxidant activity (DPPH and FRAP), caffeic acid, rosmarinic acid, morin content, and the extraction method. The second principal component is most strongly correlated with the cultivar and content of herniarin and coumarin. In Figure 5, angles below 90 °C indicate a positive correlation between the eigenvectors of variables representing antioxidant activity (FRAP and DPPH) and the vectors of variables related to the content of ferulic acid glucoside, caffeic acid, rosmarinic acid, and morin. In turn, there is a negative correlation between herniarin and coumarin content and antioxidant activity, as indicated by the obtuse angles between the eigenvectors. On the other hand, there is a positive correlation between herniarin and coumarin content and cultivar, which was confirmed by our results. The loadings of variables on the principal components (PC1 and PC2) were presented in Table S1.

**Figure 8:**
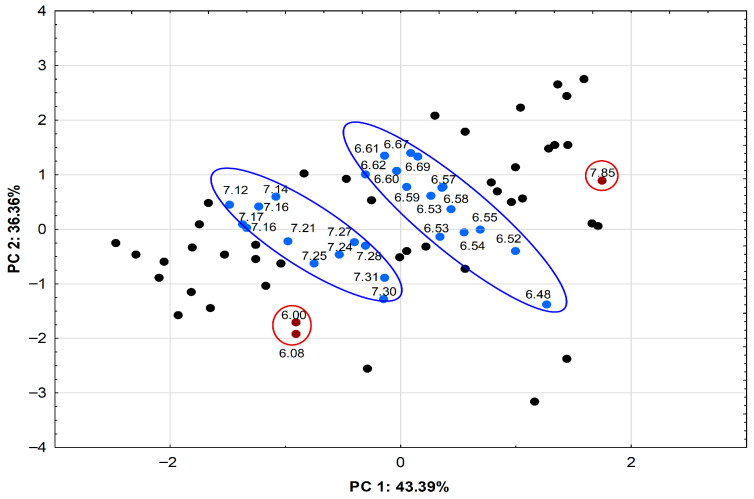
Score plot (PC 1/PC 2) of the PCA for the NMR data. Blue circles—differences between the two types of extracts, red circles—differences between cultivars.

The grouping of variables for data obtained in the aromatic range from 5.82 to 8.00 ppm of ^1^H NMR spectra for 70% MeOH extracts and CO_2_ extracts of *L*. *angustifolia* cultivar Betty’s Blue and *L*. × *intermedia* cultivar Alba is presented in Figure 8. PCA showed that the first two principal components explained 79.75% of the data variability. The first of them (PC 1) described 43.39% of the total variance, the second (PC 2) 36.36%. Two groups of variables (marked in blue), separated from the group of points marked in black, indicate differences in the presence of signals (δH = 6.48–6.70 ppm and δH = 7.12–7.30 ppm) in the two types of extracts, which was confirmed by our studies. The points marked in red represent signals at values of δH = 6.00 and 6.08 ppm occurring in the spectra recorded for CO_2_ extracts, differentiating the Betty’s Blue from Alba cultivar. However, the point marked in red (δH = 7.85 ppm) differentiates the Alba from Betty’s Blue cultivar. The loadings of the NMR variables on the principal components (PC1 and PC2) were presented in Table S2.

## 3. Materials and Methods

### 3.1. Chemicals

The reference compounds rosmarinic acid, gallic acid, caffeic acid, chlorogenic acid, morin, DPPH (2,2′-diphenyl-1-picrylhydrazyl), TPTZ (2,4,6-tripyridyl-s-triazine) and leucine-enkephalin were purchased from Sigma-Aldrich (Steinheim, Germany), while ellagic acid, trans-ferulic acid, isoquercitrin, herniarin, and apigenin were purchased from PhytoLab (Vestenbergsgreuth, Germany). Hesperidin, fisetin, formononetin, and naringenin were obtained from Carl Roth (Karlsruhe, Germany); rutin and coumarin were obtained from Acrōs Organics (Thermo Fisher Scientific, Waltham, MA, USA); and vanillin was obtained from Fluka (St. Louis, MO, USA). Methanol (99.8%), ethanol (96%), sodium carbonate, hydrochloric acid (35–38%), iron (III) chloride and Folin–Ciocalteu reagent were acquired from Avantor Performance Materials Poland S.A (Gliwice, Poland). Methanol-d4 (99.5 atom % D) was obtained from Armar Chemicals (Leipzig, Germany). HPLC-grade acetonitrile, formic acid, Trolox (6-hydroxy-2,5,7,8-tetramethylchroman-2-carboxylic acid) and purified water (Millipore Direct-Q^®^ 3UV Ultrapure (type 1) water) were purchased from Merck (Darmstadt, Germany). All reference materials and chemicals were of analytical reagent grade.

### 3.2. Plant Material

Flowers of the lavender (*Lavandula angustifolia* Mill.) cultivars Betty’s Blue, Elizabeth, Hidcote, and Blue Mountain White, and lavandin (*Lavandula* × *intermedia* Emeric ex Loisel.) cultivars Alba, Grosso, and Gros Bleu were collected at the bud stage (just before the flowering stage) in June 2019 from a plantation in central Poland. The most important criterion for selecting cultivars for the species *Lavandula angustifolia* and *Lavandula* × *intermedia* was their morphological diversity (purple-flowered cultivars: Betty’s Blue, Elizabeth, Hidcote, Gros Bleu, and Grosso, and white-flowered cultivars: Alba, Blue Mountain White), which could be associated with potential phytochemical diversity. Moreover, the plant material was obtained from a plantation that possesses a National Collection of the genus *Lavandula*. The lavender plantation is located in a dry and sunny location (latitude 52.547°, longitude 19.706°, and elevation 103 m above sea level). Throughout the year, temperatures range from −4 °C to 24 °C, but rarely fall below −14 °C or exceed 30 °C. The month with the most rain is July, with an average of 9.1 days of rain and an average rainfall of 58 mm [40]. The soil on the plantation is light, well-drained, with a pH of 6.71. The lavender is fertilized every 2–3 weeks from April to September with fertilizers designed for lavender cultivation. Ingredients such as phosphorus, potassium, nitrogen, boron, zinc, copper, manganese, molybdenum, and iron ensure lush growth and flowering. Lavender shrubs are planted 40–60 cm apart, depending on the species and cultivar. Seasonal pruning is an important maintenance practice in lavender cultivation, as it influences abundant flowering. Although *Lavandula angustifolia* and *Lavandula* × *intermedia* are considered frost-resistant species, young shrubs should be covered with white agrotextile during frosty winters.

The plant specimens were deposited in the Herbarium of the Faculty of Biology of the University of Warsaw, where they were assigned voucher numbers (Table S3). The lavender and lavandin flowers were freeze-dried for 50 h at −25 °C and 0.63 mbar (Alpha 1-2 LDplus CHRIST, Donserv, Warsaw, Poland). Lavender flowers were ground in a grinder (MMK-06M, MPM, Milanówek, Poland). The final grain size was determined using an optical microscope (SK series, OPTA-TECH, Warsaw, Poland) and was 0.1 mm. The ground samples were stored in airtight plastic containers in a dry and dark place (21 °C) until further analysis.

### 3.3. Extraction Procedure

#### 3.3.1. Ultrasound-Assisted Extraction Using 70% MeOH

The 70% methanol extracts were prepared according to the procedure described by Arceusz and Wesołowski [41] and Sytar et al. [30]. To one gram of dry, lyophilized powder, 20 mL of 70% (*v*/*v*) methanol at pH 4.0 (formic acid) was added and sonicated for 30 min in an ultrasonic bath (Sonic 5 type, 500 × 135 × 100 mm, Polsonic, Warsaw, Poland). Then, the solution was decanted, and 10 mL of 70% methanol was added to the sediment and sonicated for 15 min. This activity was repeated in the third step. The sonication process was performed at 60 °C using the following parameters: ultrasound frequency 40 kHz, and ultrasound power 160 W. The extracts were combined and centrifuged (Biomedico LC-04R, Gdynia, Poland) at 21 °C for 10 min at 1207.44× *g* (RCF), and then filtered through a qualitative filter (Whatman, grammage 80 g/m^2^, pore size 30–50 µm, Merck, Darmstadt, Germany). Three replicates were performed for each sample, and the extracts obtained were referred to as “70% MeOH extracts”.

#### 3.3.2. Supercritical CO_2_ Extraction

Supercritical CO_2_ extraction was performed using an SFE Spe-ed 4 apparatus (Applied Separations, Allentown, PA, USA) at a temperature of 55 °C and a pressure of 300 bar for 45 min [18] with a constant flow of CO_2_ of approximately 1.8 g/min. The total use of CO_2_ during a single extraction was approximately 81–85 g. The optimization parameters and extraction efficiency for the Alba cultivar were presented in Table S4. As it was shown that the use of ethanol as a co-solvent increases the yield of lavender flowers in supercritical CO_2_ extraction [42], prior to the extraction, 3 g of plant material was mixed with 10 mL of co-solvent (75% (*v*/*v*) ethanol) and placed in an extraction vessel that was thermostated for 5 min. The extraction process was carried out in duplicate for each sample, and the extracts obtained were referred to as “CO_2_ extracts”.

The 70% methanol and CO_2_ extracts were evaporated to dryness (Hei-VAP Advantage, system SC 920 G, Heidolph Instruments, Schwabach, Germany), and then dissolved in 10 mL of ultrapure water and lyophilized (Alpha 1-2 LDplus CHRIST, Donserv, Warsaw, Poland) for 50 h at −25 °C and 0.63 mbar. The extracts obtained were referred to as “dry extracts”. The samples were used for the HPLC and NMR analyses.

### 3.4. Phytochemical Profile Analysis

#### 3.4.1. Total Polyphenol Content Determination

The total polyphenolic content was assayed using the modified Waterhouse procedure [43]. Firstly, 15 µL of extract, 1185 µL of ultrapure water, and 75 µL of Folin–Ciocalteu reagent were added to an Eppendorf tube and vortexed (Vortex DragonLab MX-S, Józefów, Poland) for 2 min. Then, 225 µL of 20% (*w*/*v*) Na_2_CO_3_ was added, and the sample was mixed and heated (Digital Heatblock, VWR, Gdańsk, Poland) at 40 °C for 20 min. The absorbance of the sample was measured using an Evolution 60S UV–visible spectrophotometer (Thermo Scientific, Waltham, MA, USA) at a wavelength of λ = 765 nm against a blank prepared by replacing the extract with ultrapure water. Three replicates were performed for each sample. The total polyphenol content was expressed as gallic acid equivalents (GAE) in milligrams per gram of dry plant matter [mg_GAE/g_d.m.] based on the standard curve y = 0.0011x + 0.0016, where y is the absorbance (the optical path length was 1 cm), and x is the concentration of gallic acid in mg/L (R^2^ = 0.999).

#### 3.4.2. HPLC-DAD

##### Extract Analysis

The separation of polyphenolic compounds was performed using a Hitachi Chromaster system (Tokyo, Japan) equipped with UV/VIS diode array detector (5430), a column oven (5310), an autosampler (5260), and a pump (5160). The freeze-dried extract (40 mg) was dissolved in 2 mL of the solvent that was previously used for extraction, filtered through a syringe filter (0.45 μm, PTFE–polytetrafluoroethylene, Bionovo, Emeryville, CA, USA), and transferred to vials, which were then placed in an autosampler. The mobile phase was composed of eluent A (0.1% (*v*/*v*) formic acid in water) and eluent B (0.1% (*v*/*v*) formic acid in acetonitrile). The following linear gradient was used for the separation of compounds: 90–80% A (0–35 min), 80–65% A (35–60 min), 65–90% A (60–60.1 min), and 90% A (60.1–70 min) at a flow rate of 1 mL/min at 30 °C. During the analysis, 20 μL of the sample was applied to the Purospher STAR RP-18e column (particle size: 5 μm; 250 × 4.6 mm; Merck, Darmstadt, Germany) using an autosampler. The HPLC analysis of each sample was performed in triplicate. The chromatograms were recorded at wavelengths (λ) of 250, 280, and 330 nm. The separated compounds were identified by comparing their UV-Vis spectra and retention times with those of standard substances.

##### Validation of the HPLC Results

The concentrations of the compounds present in the lavender and lavandin extracts were determined using standard curves (Table S5). One milligram of the standards (caffeic acid, rosmarinic acid, ferulic acid, morin, coumarin, and herniarin) was dissolved in 1 mL of methanol, and then the solution was mixed and filtered through a syringe filter (0.45 µm, Bionovo, Poland). HPLC analysis of the standard substances was performed under the same conditions as for the tested extracts. Three replicates were performed for each sample.

The linearity, limit of detection (LOD), and limit of quantification (LOQ) were determined from the standard curve to validate the procedure. The linearity was assessed by calculating the value of R^2^ (the coefficient of determination) of the standard curve obtained for each standard substance. The LOD values were obtained using the following formula: LOD = 3.3 × σ/S. The LOQ values were calculated using the following formula: LOQ =10 × σ/S, where S is the slope of the calibration curve, and σ is the standard deviation of the y-intercept. The values of the coefficient of determination (R^2^) for the standard curves were higher than 0.998, indicating high linearity. For the analysed substances, the LOD values ranged from 7 to 26 mg/L, whereas the LOQ values ranged from 20 to 79 mg/L, indicating that the applied method was suitable for quantifying the individual compounds (Table S5).

Three different concentrations (0.10, 0.40, and 0.80 mg/mL) of the compounds with the highest abundance (rosmarinic acid and herniarin) were used to determine the intraday and interday precision (Table S6). The analysis was performed in triplicate on the same day and the next day. The RSD (relative standard deviation) values below 1% indicated that the method had high repeatability.

#### 3.4.3. Determination of the Polyphenolic Compound Profile Using UHPLC/HRMS

To confirm the identity of the compounds present in the lavender extracts, UHPLC/HRMS (ultra-high-performance liquid chromatography/high-resolution mass spectrometry) analysis was used. The analysis was performed using an ACQUITY UPLC I-Class (Waters, Milford, MA, USA) coupled to a Synapt G2-S mass spectrometer (Waters, Milford, MA, USA) with an electrospray ion source and a TOF (time-of-flight) analyser. The resolution of the TOF analyser was 40,000 FWHM. The mobile phase was composed of 0.1% formic acid (*v*/*v*) in water (eluent A) and methanol (eluent B). The compounds were separated using a gradient of 95% A (0–3 min), 95–0% A (3–30 min), 0–95% A (30–33 min), and 95% A (33–35 min) at a flow rate of 0.30 mL/min. During the analysis, 2 µL of the sample was applied to an Acquity UPLC BEH C18 column (particle size: 1.7 µm; 2.1 × 100 mm; Waters, Milford, MA, USA) at 40 °C. Detection was performed at λ = 254 and 280 nm. The mass spectrometry measurements were performed in both negative and positive ion modes. The measurements were conducted with the capillary voltage set at 3.05 kV in negative ion mode and 2.94 kV in positive ion mode. The desolvation gas (nitrogen) flow and temperature in negative ion mode were 774 L/h and 350 °C, while in positive ion mode, they were 799 L/h and 450 °C. A leucine-enkephalin solution was applied as the Lock-Spray standard substance. The MassLynx V4.1 software package (Waters, Milford, MA, USA) was used to process the data. The MassBank commercial library and NIST Chemistry WebBook were employed to identify the compounds.

#### 3.4.4. NMR Analysis

One-dimensional (^1^H NMR) and two-dimensional (COSY, HSQC, HMBC) spectra for the 70% MeOH and CO_2_ extracts were recorded using a Bruker Avance Neo 400 NMR and Avance III 500 NMR spectrometer (11.7 T, Billerica, MA, USA). Briefly, 30 mg of the lyophilized extracts were dissolved in 1.25 mL of deuterated methanol, and the samples were sonicated (Sonic 2 type, 150 × 135 × 100 mm, Polsonic, Warsaw, Poland) for an hour. The sonication process was performed at 21 °C using the following parameters: ultrasound frequency 40 kHz, and ultrasound power 50 W. Then the samples were centrifuged (MiniStar, VWR, Gdańsk, Poland) at 21 °C for 10 min at 55.9× *g* (RCF). The one-dimensional spectra for the standard substances (caffeic acid, rosmarinic acid, morin, and herniarin) were recorded using a Varian VNMRS 300 Oxford spectrometer (Palo Alto, CA, USA). A 1 mL volume of deuterated methanol was mixed with 10 mg of the pure substances. The spectra of the extracts and standards were analysed using the MestReNova 11.0 software (Mestrelab Research, Santiago de Compostela, Spain).

### 3.5. Antioxidant Activity

#### 3.5.1. FRAP Assay

The antioxidant activity was determined by FRAP assay according to the modified method described by Benzie and Strain [44]. Before analysis, the extracts were diluted 50-fold with water. The FRAP reagent was prepared by mixing 300 mM acetate buffer, pH 3.6, 10 mM TPTZ solution, with 40 mM HCl as a solvent, and 20 mM iron (III) chloride solution in a 10:1:1 (*v*/*v*/*v*) ratio. Firstly, 50 μL of the diluted extract and 1000 μL of the FRAP reagent were placed in an Eppendorf tube. Then, the reaction mixture was mixed (Vortex DragonLab MX-S, Józefów, Poland) and thermostatted (Digital Heatblock, VWR, Gdańsk, Poland) at 37 °C for 4 min. The absorbance of the sample was measured using an Evolution 60S UV–visible spectrophotometer (Thermo Scientific, Waltham, MA, USA) at a wavelength of λ = 593 nm against the blank solution. Three replicates were performed for each extract. The antioxidant activity was expressed as Trolox equivalents (TE) in micromoles per gram of dry plant matter [µmol_TE/g_d.m.] based on the standard curve y = 0.0021x − 0.0108, where y is the absorbance (the optical path length was 1 cm), and x is the Trolox concentration in µmol/L (R^2^ = 0.998).

#### 3.5.2. DPPH Assay (Electron Paramagnetic Resonance Test)

The antioxidant activity was determined by DPPH assay according to the procedure described by Sanna et al. [45]. The DPPH solution was prepared by dissolving 12.5 mg of DPPH in 25 mL of methanol. 20 μL of the sample and 180 μL of DPPH solution were placed in an Eppendorf tube. A blank was prepared by replacing the extract with the solvent. The samples were mixed and left in the dark at 21 °C for 30 min. EPR measurements were carried out in 50 μL hematocrit capillaries (IntraMark, Blaubrand, Wertheim, Germany) using a MiniScope MS200 spectrometer (Magnettech GmbH, Berlin, Germany) at room temperature (21 °C). The analyses were performed using the following parameters: central field 330 mT, microwave power 6 mW, modulation amplitude 0.10 mT, sweep range 9.9 mT, and measurement duration 20 s. Three replicates were performed for each extract. The antioxidant activity was expressed as Trolox equivalents (TE) in micromoles per gram of dry plant matter [µmol_TE/g_d.m.] based on the standard curve y = 1.22x − 0.12, where y [AU] is spectrum intensity (measured as the area under the absorption curve obtained by double integration of the obtained derivative spectrum with the use of MultiPlot software (1.0.0.486) (Magnettech GmbH, Berlin, Germany), and x is the Trolox concentration in µmol/mL (R^2^ = 0.999).

### 3.6. Statistical Analysis

Statistical analysis was performed using TIBCO Statistica 13.3 (StatSoft Poland). The Shapiro–Wilk test was used to verify the normality of the data for the groups, while the Brown–Forsythe test was used to verify the homogeneity of the variance. One-way analysis of variance (ANOVA) combined with the Tukey post hoc RIR test was used to determine whether there were significant differences between samples (*p* < 0.05). Multiple regression analysis was used to assess the relationship between the antioxidant activity of extracts and the content of polyphenolic compounds with a probability of 5%. The obtained results were also subjected to principal component analysis (PCA) for the HPLC and antioxidant data, and separately for the NMR data.

## 4. Conclusions

In this study, a range of analytical techniques was used to identify compounds present in both lavender and lavandin extracts. The total polyphenol content was determined using UV-VIS absorption spectroscopy, while the content of selected compounds was determined using HPLC-DAD. The ^1^H NMR analysis revealed the presence of characteristic signals that could differentiate between cultivars of different species. In the aliphatic region, a doublet originating from the protons of the alanine methyl group was only present in the spectra of the Betty’s Blue cultivar, while in the aromatic region, a doublet at δH = 7.85 ppm was only present in the spectra of the Alba cultivar. The signals present in both cultivars that could distinguish between the 70% MeOH and CO_2_ extracts originated from the protons of threonine and pyruvic acid and the protons of the -(CH_2_)n- group of palmitic acid. Furthermore, the ^1^H NMR analysis showed that the spectra recorded for the 70% MeOH extracts had significantly more resonance signals than those for the CO_2_ extracts due to the presence of characteristic signals for the protons of hydrophilic compounds. On the other hand, the CO_2_ extracts were characterized by signals from lipophilic compounds (coumarin, herniarin, and fatty acids).

The HPLC-DAD analysis of the extracts of *L*. *angustifolia* revealed higher rosmarinic acid, ferulic acid glucoside, morin, and caffeic acid contents, whereas the extracts of *L*. × *intermedia* exhibited higher concentrations of coumarin and herniarin. The compounds present at high concentrations in the extracts could be potential markers for differentiating between cultivars. These potential markers are rosmarinic acid and morin for the Betty’s Blue cultivar; rosmarinic acid and ferulic acid glucoside for the Elizabeth and Hidcote cultivars; ferulic acid glucoside for the Blue Mountain White cultivar; and coumarin and herniarin for the Alba, Grosso, and Gros Bleu cultivars.

For all the analysed lavender and lavandin cultivars, ultrasound-assisted extraction with 70% MeOH was identified as the most effective method for extracting polyphenolic compounds with antioxidant activity (Table S7). These compounds were also present in the CO_2_ extracts but their content was only 10% of the content in the 70% MeOH extracts. Furthermore, for all cultivars (except Gros Bleu), the CO_2_ extracts were found to contain higher levels of lipophilic compounds, namely coumarin and herniarin, with levels almost twice as high as those observed in the ultrasound-assisted extracts.

In recent years, there has been increasing interest in natural products among consumers. The utilization of plant extracts has become increasingly prevalent in many sectors, including pharmaceuticals, cosmetics, food, and agriculture. Therefore, expanding our knowledge on the metabolic profiles of *L. angustifolia* and *L*. × *intermedia* cultivars is of great importance from both a scientific and practical perspective. The employment of the ^1^H NMR technique allows for the acquisition of a compound profile, which could serve as the basis for identifying cultivars of different species. The analysis of the chemical composition, in conjunction with the elucidation of the mechanism of action of plant secondary metabolites, will enable the confirmation of the beneficial biological properties of plant raw materials, including lavender and lavandin.

As different phenolic compositions can influence the action of plant extract applied either as a cosmetic or as a nutraceutical preparation, the method of identifying cultivars with higher amount of phenolics from a selected class would enable better quality control in such products. Flavonols like morin have been proposed to support the anti-acne therapy [46] and morin itself was proposed as a promising adjuvant in the therapy of neurological diseases [47]. Moreover, rosmarinic acid was shown to decrease pH of skin surface and improve the functioning of skin barrier [48], and it was proposed as an adjuvant in the therapy of cancer, diabetes and neurodegenerative disorders [49], while coumarins were shown to act as antioxidants and photoprotective agents [50]. However, to this end, the assessment of stability of metabolome of different cultivars of *L. angustifolia* and *L*. × *intermedia* should be performed by checking the influence of various cultivation conditions, i.e., different soil or microclimate as well as the inter-season variability.

## Figures and Tables

**Table 1:** Total polyphenol content in *L. angustifolia* and *L*. × *intermedia* extracts obtained using different extraction methods.

Species	Cultivar	70% MeOH Extract [mg_GAE/g_d.m.]	CO_2_ Extract [mg_GAE/g_d.m.]
*L*. *angustifolia*	Betty’s Blue	39 ± 1.2 ^a^	4.2 ± 0.14 ^b^
	Elizabeth	38.7 ± 0.26 ^a^	3.95 ± 0.043 ^b^
	Hidcote	25.4 ± 0.40 ^a^	1.45 ± 0.042 ^b^
	BMW	27.5 ± 0.40 ^a^	2.20 ± 0.070 ^b^
*L*. × *intermedia*	Alba	34.3 ± 1.4 ^a^	2.58 ± 0.080 ^b^
	Grosso	30 ± 1.3 ^a^	2.06 ± 0.032 ^b^
	Gros Bleu	32 ± 1.0 ^a^	1.34 ± 0.023 ^b^

BMW—Blue Mountain White. Letters (^a^, ^b^) indicate statistically significant differences between extraction methods at the level of *p* < 0.05 according to ANOVA. Data represent the mean ± standard deviation (SD), (*n* = 3).

**Table 2:** Content of polyphenolic compounds present in 70% MeOH and CO_2_ extracts of different *L*. *angustifolia* and *L*. × *intermedia* cultivars.

Extraction Method	Cultivar	Caffeic Acid	Ferulic Acid Glucoside *	Rosmarinic Acid	Morin	Coumarin	Herniarin
		[mg/g_d.e.]					
70% MeOH extract	Betty’s Blue	2.30 ± 0.010 ^a^	4.33 ± 0.060 ^b^	14.1 ± 0.34 ^a^	9.2 ± 0.30 ^a^	2.70 ± 0.050 ^b^	2.74 ± 0.070 ^b^
	Elizabeth	3.02 ± 0.051 ^a^	7.7 ± 0.50 ^ab^	11.0 ± 0.90 ^a^	5.8 ± 0.52 ^ab^	3.0 ± 0.35 ^b^	3.1 ± 0.33 ^b^
	Hidcote	2.85 ± 0.060 ^a^	7.6 ± 0.54 ^ab^	8.5 ± 0.12 ^ab^	2.95 ± 0.051 ^b^	2.7 ± 0.10 ^b^	3.6 ± 0.21 ^b^
	BMW	2.1 ± 0.14 ^a^	9.1 ± 0.43 ^a^	5.0 ± 0.14 ^b^	3.21 ± 0.020 ^b^	2.7 ± 0.24 ^b^	3.4 ± 0.22 ^b^
	Alba	2.1 ± 0.10 ^a^	4.9 ± 0.20 ^b^	4.5 ± 0.16 ^b^	2.2 ± 0.13 ^b^	8.3 ± 0.22 ^a^	7.1 ± 0.30 ^a^
	Grosso	2.76 ± 0.011 ^a^	6.95 ± 0.084 ^ab^	4.7 ± 0.40 ^b^	3.53 ± 0.080 ^b^	6.6 ± 0.26 ^a^	8.9 ± 0.16 ^a^
	Gros Bleu	2.09 ± 0.083 ^a^	3.77 ± 0.072 ^b^	3.9 ± 0.80 ^b^	3.1 ± 0.23 ^b^	5.9 ± 0.10 ^a^	6.8 ± 0.30 ^a^
CO_2_ extract	Betty’s Blue	1.03 ± 0.013 ^a^	2.66 ± 0.010 ^a^	4.1 ± 0.21 ^a^	3.2 ± 0.16 ^a^	5.4 ± 0.20 ^bc^	6.6 ± 0.10 ^b^
	Elizabeth	1.83 ± 0.034 ^a^	4.6 ± 0.20 ^a^	4.1 ± 0.20 ^a^	2.05 ± 0.071 ^a^	8.1 ± 0.12 ^b^	7.1 ± 0.10 ^b^
	Hidcote	0.60 ± 0.030 ^a^	1.51 ± 0.010 ^a^	1.26 ± 0.020 ^a^	0.59 ± 0.030 ^a^	6.95 ± 0.040 ^b^	8.1 ± 0.16 ^b^
	BMW	0.857 ± 0.0021 ^a^	4.60 ± 0.014 ^a^	1.3 ± 0.25 ^a^	0.6 ± 0.13 ^a^	9.6 ± 0.10 ^b^	7.4 ± 0.10 ^b^
	Alba	0.470 ± 0.0014 ^a^	1.75 ± 0.020 ^a^	1.13 ± 0.080 ^a^	0.60 ± 0.050 ^a^	16.92 ± 0.080 ^a^	13.38 ± 0.053 ^a^
	Grosso	0.75 ± 0.010 ^a^	2.62 ± 0.063 ^a^	1.34 ± 0.030 ^a^	0.930 ± 0.0010 ^a^	15.00 ± 0.010 ^a^	12.54 ± 0.022 ^a^
	Gros Bleu	n.d.	n.d.	n.d.	n.d.	2.56 ± 0.010 ^c^	2.07 ± 0.050 ^c^

BMW—Blue Mountain White; n.d.—no data. * Ferulic acid glucoside content is expressed in ferulic acid equivalents. Data represent the mean ± standard deviation (SD), (*n* = 3). Different letters (^a^, ^b^, ^c^) indicate statistically significant differences between cultivars at the level of *p* < 0.05 according to ANOVA.

**Table 3:** Antioxidant activity determined by FRAP and DPPH assays for *L. angustifolia* and *L*. × *intermedia* extracts obtained using different extraction methods.

Species	Cultivar	FRAP [µmol_TE/g_d.m.]		DPPH [µmol_TE/g_d.m.]	
		70% MeOH Extract	CO_2_ Extract	70% MeOH Extract	CO_2_ Extract
*L*. *angustifolia*	Betty’s Blue	231 ± 1.5 ^a^	17.5 ± 0.22 ^b^	238 ± 1.9 ^a^	20.5 ± 0.43 ^b^
	Elizabeth	227 ± 4.0 ^a^	15.8 ± 0.35 ^b^	233 ± 6.0 ^a^	19.8 ± 0.21 ^b^
	Hidcote	144 ± 1.8 ^a^	3.8 ± 0.32 ^b^	160 ± 10 ^a^	3.88 ± 0.030 ^b^
	BMW	184 ± 1.5 ^a^	7.4 ± 0.53 ^b^	186 ± 5.4 ^a^	7.00 ± 0.050 ^b^
*L*. × *intermedia*	Alba	231 ± 1.8 ^a^	7.1 ± 0.22 ^b^	236 ± 7.0 ^a^	7.22 ± 0.090 ^b^
	Grosso	196 ± 2.6 ^a^	5.7 ± 0.13 ^b^	187 ± 7.0 ^a^	7.10 ± 0.050 ^b^
	Gros Bleu	205 ± 1.0 ^a^	1.42 ± 0.080 ^b^	196 ± 8,0 ^a^	1.42 ± 0.040 ^b^

BMW—Blue Mountain White. Data represent the mean ± standard deviation (SD), (*n* = 3). Letters (^a^, ^b^) indicate statistically significant differences between extraction methods at the level of *p* < 0.05 according to ANOVA.

## Data Availability

The original contributions presented in this study are included in the article/Supplementary Materials. Further inquiries can be directed to the corresponding author.

## Supplementary Materials

The following supporting information can be downloaded at: https://www.mdpi.com/article/10.3390/plants15020217/s1, Figure S1: HPLC chromatogram for the standard mixture recorded at λ = 330 nm; Figure S2: ^1^H NMR spectra in the range from 0.50 to 8.50 ppm for 70% MeOH extracts (A) and CO_2_ extracts (B) of *L*. *angustifolia* (Betty’s Blue (1), Elizabeth (2), Hidcote (3), and Blue Mountain White (4)) and *L*. × *intermedia* (Alba (5), Grosso (6), and Gros Bleu (7)) cultivars. Green frame—aliphatic region; red frame—sugar region; blue frame—aromatic region; Table S1: The loadings of variables on the principal components (PC1 and PC2); Table S2: The loadings of the NMR variables on the principal components (PC1 and PC2); Table S3: Voucher numbers for *L*. *angustifolia* and *L*. × *intermedia* cultivars; Table S4: The optimization parameters and extraction efficiency SFE for the Alba cultivar; Table S5: Equations of calibration curves, linear range, LOD, and LOQ for compounds found in *L*. *angustifolia* and *L*. × *intermedia*; Table S6: Intraday and interday precision for selected reference substances; Table S7: Extraction efficiency for both methods for all cultivars.

## Abbreviations

The following abbreviations are used in this manuscript:

BMW	Blue Mountain White cultivar
DPPH	2,2′-diphenyl-1-picrylhydrazyl
EPR	Electron Paramagnetic Resonance
FRAP	Ferric Reducing Antioxidant Power
HMDB	Human Metabolome Database
HPLC	High-Performance Liquid Chromatography
HRMS	High Resolution Mass Spectrometry
LOD	Limit of Detection
LOQ	Limit of Quantification
NMR	Nuclear Magnetic Resonance
PCA	Principal Component Analysis
SFE	Supercritical Fluid Extraction
TE	Trolox Equivalents
TPTZ	2,4,6-tripyridyl-s-triazine
UAE	Ultrasound-Assisted Extraction
UHPLC	Ultra-High-Performance Liquid Chromatography
