# Supplementary material for: Application of 1H NMR and HPLC-DAD in Metabolic Profiling of Extracts of Lavandula angustifolia and Lavandula × intermedia Cultivars
Source: Plants (Basel). 2026 Jan 10;15(2):217. doi: 10.3390/plants15020217 (PMC12844681; doi:10.3390/plants15020217)
Supplement: Supplementary file 1 [file plants-15-00217-s001.zip › plants-4045001-supplementary.pdf]

Supplementary Materials

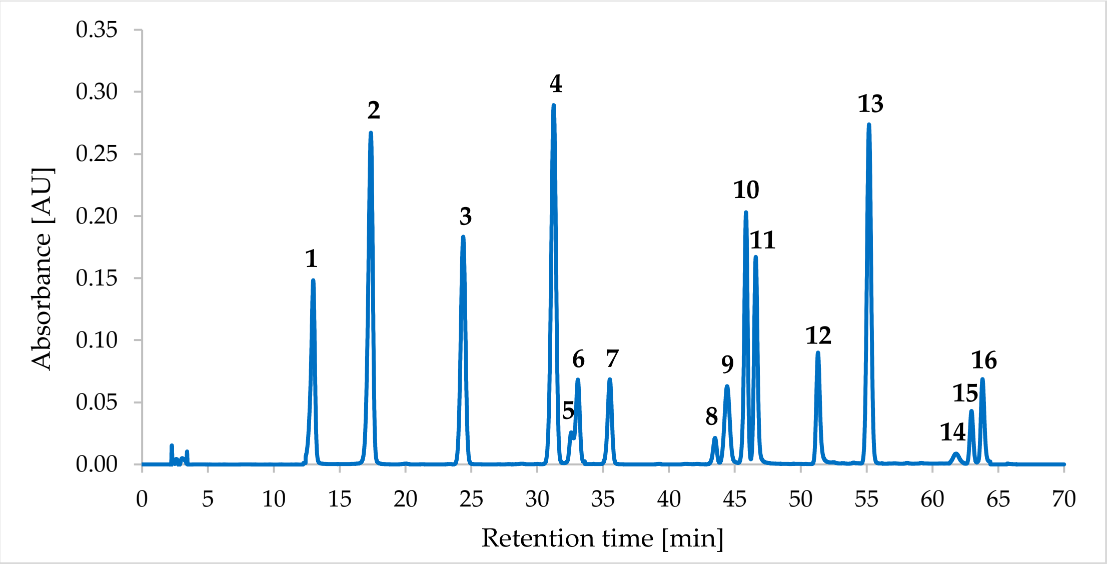

Figure S1. HPLC chromatogram for the standard mixture recorded at λ = 330 nm

Numbering of polyphenolic compounds: 1 – chlorogenic acid, 2 – caffeic acid, 3 – vanillin, 4 – ferulic acid, 5 – ellagic acid, 6 – rutin, 7 – isoquercitrin, 8 – hesperidin, 9 – coumarin, 10 – rosmarinic acid, 11 – fisetin, 12 – morin, 13 – herniarin, 14 – formononetin, 15 – naringenin, 16 – apigenin

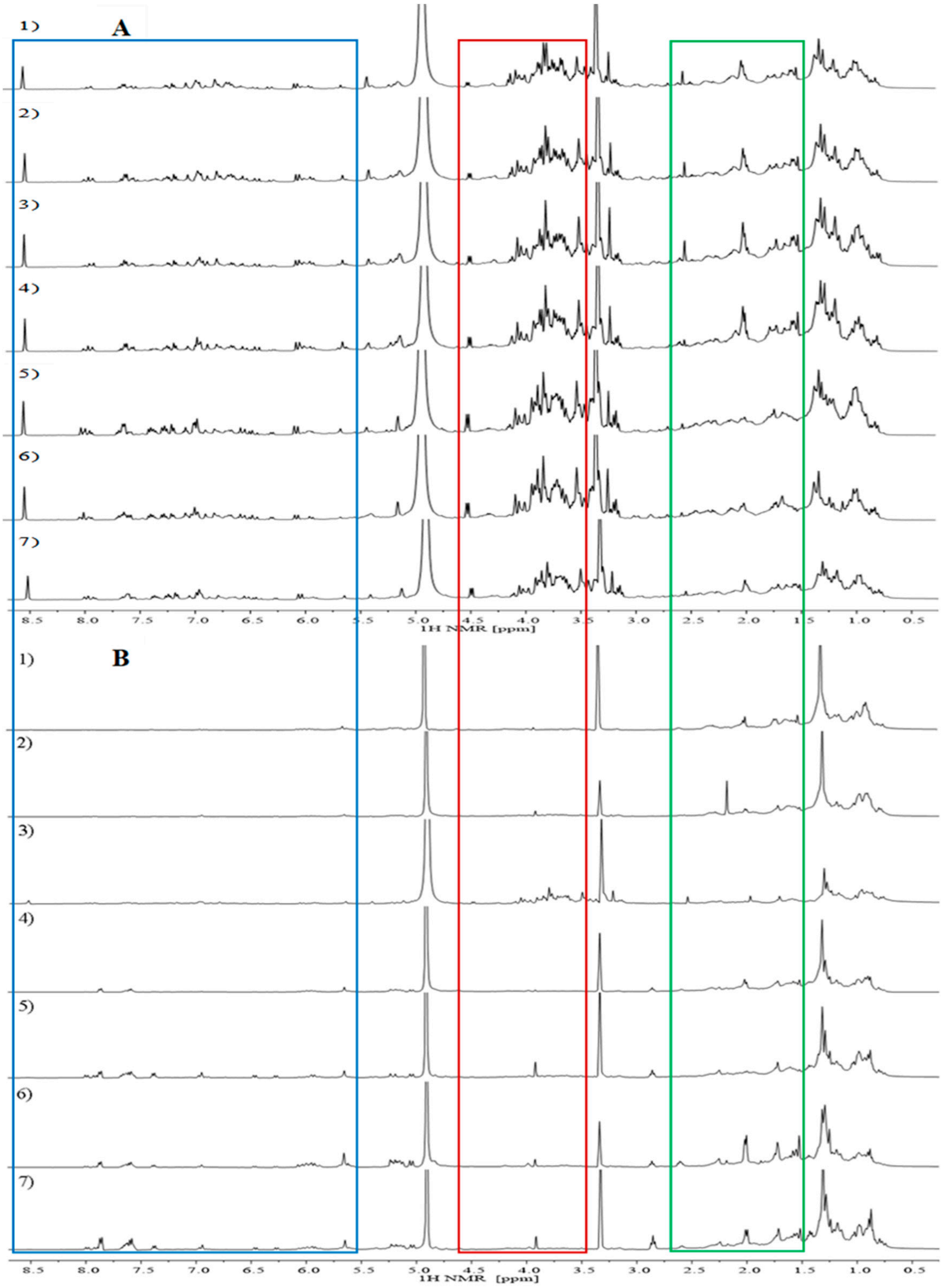

Figure S2. ^{1}H NMR spectra in the range from 0.50 to 8.50 ppm for 70% MeOH extracts (A) and CO_2 extracts (B) of *L. angustifolia* (Betty's Blue (1), Elizabeth (2), Hidcote (3), and Blue Mountain White (4)) and *L.* x *intermedia* (Alba (5), Grosso (6), and Gros Bleu (7)) cultivars. Green frame—aliphatic region; red frame—sugar region; blue frame—aromatic region.

Table S1. The loadings of variables on the principal components (PC1 and PC2)

Variables	PC1	PC2
Extraction method	**-0.913811**	0.371427
Cultivar	-0.356850	**-0.747155**
FRAP	**0.914688**	-0.344734
DPPH	**0.928445**	-0.314240
Caffeic acid	**0.910590**	-0.208979
Ferulic acid glucoside	0.769246	-0.217854
Rosmarinic acid	**0.897372**	0.246652
Morin	**0.848981**	0.229917
Coumarin	-0.696283	**-0.430084**
Herniarin	-0.646437	**-0.497733**

Table S2. The loadings of the NMR variables on the principal components (PC1 and PC2)

Variables	PC1	PC2
ppm	**0.908842**	0.381855
Hz	**0.908847**	0.381839
Width	-0.001904	0.574920
Area	0.597106	-0.594369

Table S3. Voucher numbers for *L. angustifolia* and *L.* x *intermedia* cultivars

Species	Cultivar	Voucher number
Lavandula angustifolia	Betty's Blue	WA0000111926
	Elizabeth	WA0000111925
	Hidcote	WA0000111924
	Blue Mountain White	WA0000162742
Lavandula x *intermedia*	Alba	WA0000162743
	Grosso	WA0000111928
	Gros Bleu	WA0000111927

Table S4. The optimization parameters and extraction efficiency SFE for the Alba cultivar.

Method	Temperature [°C]	Pressure [bar]	Extraction time [min]	Extraction efficiency [%]
1	40	200	45	4.92
2	55	300	45	6.40
3	50	332	60	6.01

Table S5. Equations of calibration curves, linear range, LOD and LOQ for compounds found in *L. angustifolia* and *L.* x *intermedia*

Chemical compound	Retention time [min]	Retention interval [min]	Calibration curve y [AU], x [mg/mL]	R^2	Linear range [mg/L]	LOD [mg/L]	LOQ [mg/L]
Caffeic acid	17.5	16.65 – 18.34	y = 49472514.87x + 2055.42	0.999	10 – 120	7	21
Ferulic acid	31.1	30.86 – 31.29	y = 50249746.06x + 54989.28	1.000	10 – 600	10	30
Coumarin	44.1	43.64 – 44.63	y = 12371528.31x + 65990.61	0.999	10 – 400	7	20
Rosmarinic acid	45.9	45.05 – 46.68	y = 28614208.85x – 9260.92	0.998	10 – 400	26	79
Morin	51.2	50.63 – 51.82	y = 15139387.44x – 43686.62	0.999	10 – 400	21	64
Herniarin	55.0	54.15 – 55.91	y = 44802946.76x + 160450.31	0.999	10 – 300	20	61

Table S6. Intraday and interday precision for selected reference substances

Reference substance	Concentration of the reference substance [mg/mL]	Intraday precision RSD [%]	Interday precision RSD [%]
Rosmarinic acid	0.10	0.313	0.179
	0.40	0.302	0.266
	0.80	0.328	0.213
Herniarin	0.10	0.087	0.046
	0.40	0.170	0.203
	0.80	0.152	0.204

Table S7. Extraction efficiency for both methods for all cultivars

**Cultivar**	**Extraction efficiency** [%]	
	**70% MeOH extract**	**CO_2 extract**
Betty's Blue	20.84	6.89
Elizabeth	20.34	4.36
Hidcote	17.26	2.90
BMW	18.70	4.64
Alba	20.40	6.40
Grosso	16.86	3.89
Gros Bleu	16.58	3.43
